# Leakage-aware pre-event machine learning evaluation for horse-race prediction under temporal validation

**DOI:** 10.3389/frai.2026.1896192

**Published:** 2026-09-24

**Authors:** Shuichi Sugiura

**Affiliations:** Graduate School of Psychology, Kansai University, Suita, Japan

**Keywords:** data leakage, feature evaluation, horse-race prediction, machine learning, model evaluation, predictive modeling, prospective prediction, temporal validation

## Abstract

Horse-racing prediction was used as a case study to develop and evaluate a leakage-aware machine-learning framework based on information available after race-entry finalization and before outcomes were known. Japanese Racing Association flat-race data obtained through JRA-VAN Data Lab. and exported using TARGET frontier JV were split temporally: 2015–2022 for training, 2023–2024 for validation, and January 5, 2025 to May 10, 2026 for independent testing. The test set contained 63,910 horse-level observations from 4,556 races. Confidence intervals were estimated using 10,000 race-level bootstrap resamples. Two horse-level binary outcome labels were evaluated: a win outcome and a JRA place-rule-compatible place outcome derived from the processed field_size variable. Compared with the augmented current_full_model, the hyperparameter-matched no-theory sensitivity-analysis model matched_no_theory_model performed better for both outcomes. For the win outcome, ROC AUC was 0.7543 (95% CI: 0.7475–0.7609) for matched_no_theory_model and 0.7293 (95% CI: 0.7224–0.7362) for current_full_model. For the JRA place-rule-compatible place outcome, ROC AUC was 0.7513 (95% CI: 0.7469–0.7558) and 0.7164 (95% CI: 0.7118–0.7212), respectively. An exploratory race-level uncertainty diagnostic was evaluated separately and was not incorporated into the horse-level prediction models. These findings indicate that the evaluated theory-score block did not improve temporal test-set performance. Domain-derived feature blocks should be evaluated incrementally through leakage audits, temporal validation, feature-block ablation, and matched sensitivity analyses before deployment.

## Graphical Abstract

**Figure:**
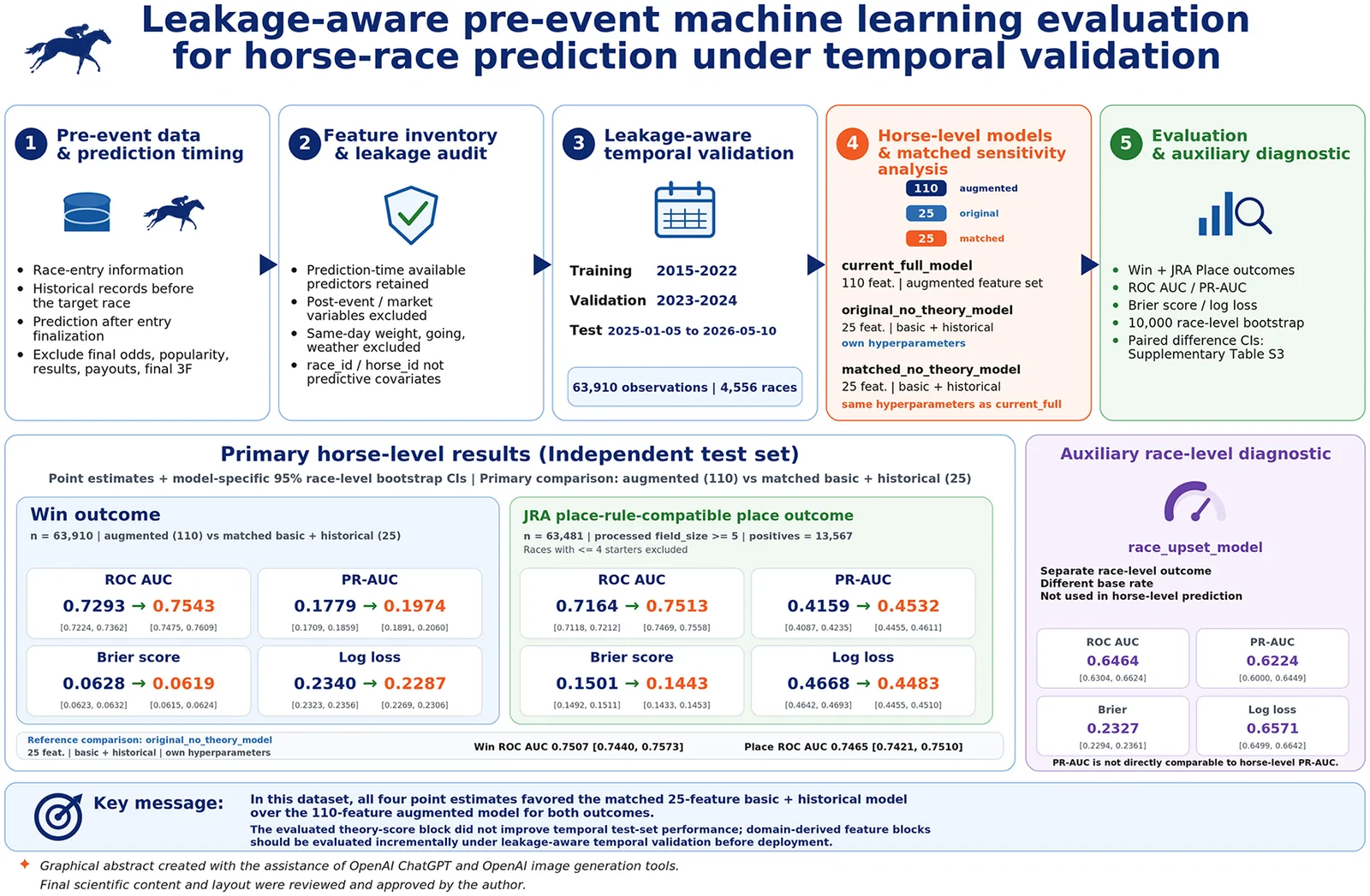

## 1 Introduction

High discriminatory performance alone is insufficient for machine-learning models intended for prospective prediction. It is also necessary to ensure that the reported performance is based exclusively on information available at the time a prediction is made. When pre-event information and post-event information coexist within the same database, information observed after the target event may inadvertently enter feature engineering, preprocessing, model selection, or evaluation procedures, resulting in overly optimistic performance estimates. Such data leakage has been defined as the incorporation of information that is available during model development but unavailable in real-world deployment into predictive models (Kaufman et al., 2012). In addition, for data with temporal dependence, random data partitioning may overestimate independence among observations and lead to optimistic estimates of future generalization performance (Roberts et al., 2017). Therefore, prospective prediction models should explicitly account for prediction-time information availability, leakage prevention, and temporal validation.

Horse-racing prediction provides a useful case study for examining these issues. Horse-racing databases typically contain both pre-event information, such as racecourse, surface type, distance, draw position, jockey, trainer, pedigree, and historical performance records, and post-event information, such as finishing position, payouts, final odds, and popularity rankings. Quantitative prediction models have long been applied to horse-racing prediction (Bolton and Chapman, 1986; Lessmann et al., 2009), and machine-learning approaches have recently become increasingly common in sports outcome prediction more broadly (Bunker and Thabtah, 2019; Horvat and Job, 2020). However, evaluation frameworks that explicitly incorporate leakage audits based on prediction-time availability and temporal validation using future data remain limited.

Recent guidance for prediction model reporting has emphasized the importance of clearly defining the prediction target, prediction timing, candidate predictors, outcome definitions, validation strategies, and performance measures (Collins et al., 2024). In the present study, these transparency principles were used as a general reference framework to conduct a feature inventory and leakage audit. Furthermore, because feature design and feature selection are known to substantially influence predictive performance (Guyon and Elisseeff, 2003), explicit comparisons were conducted between domain-derived feature blocks and simpler historical feature sets.

In this manuscript, the theory-score block refers to a set of aggregate and domain-derived feature blocks, including jockey and sire aggregate features, same-course features, maiden-venue features, and rule-based flags. These features were evaluated as historical aggregate or domain-rule feature blocks rather than as evidence that domain knowledge is generally useful or harmful.

The objective of this study was to develop and evaluate a leakage-aware machine-learning evaluation framework for horse-racing prediction using only information considered available after race-entry finalization and before race outcomes were known. The novelty of the study lies in integrating prediction-time information availability, feature inventory construction, leakage auditing, temporal validation, feature-block comparison, matched sensitivity analysis, and race-level bootstrap uncertainty estimation into a single deployment-oriented evaluation framework. Specifically, current_full_model, original_no_theory_model, and matched_no_theory_model were compared using the same temporally separated test set. In addition, race_upset_model was independently evaluated as an exploratory and auxiliary race-level uncertainty diagnostic. This study was not intended to assess the general value of domain knowledge itself; rather, it examined whether the currently implemented theory-score block provided incremental predictive value beyond a simpler and deployment-oriented feature set.

Figure 1 summarizes the leakage-aware pre-event machine learning evaluation pipeline used in this study.

**Figure 1:**
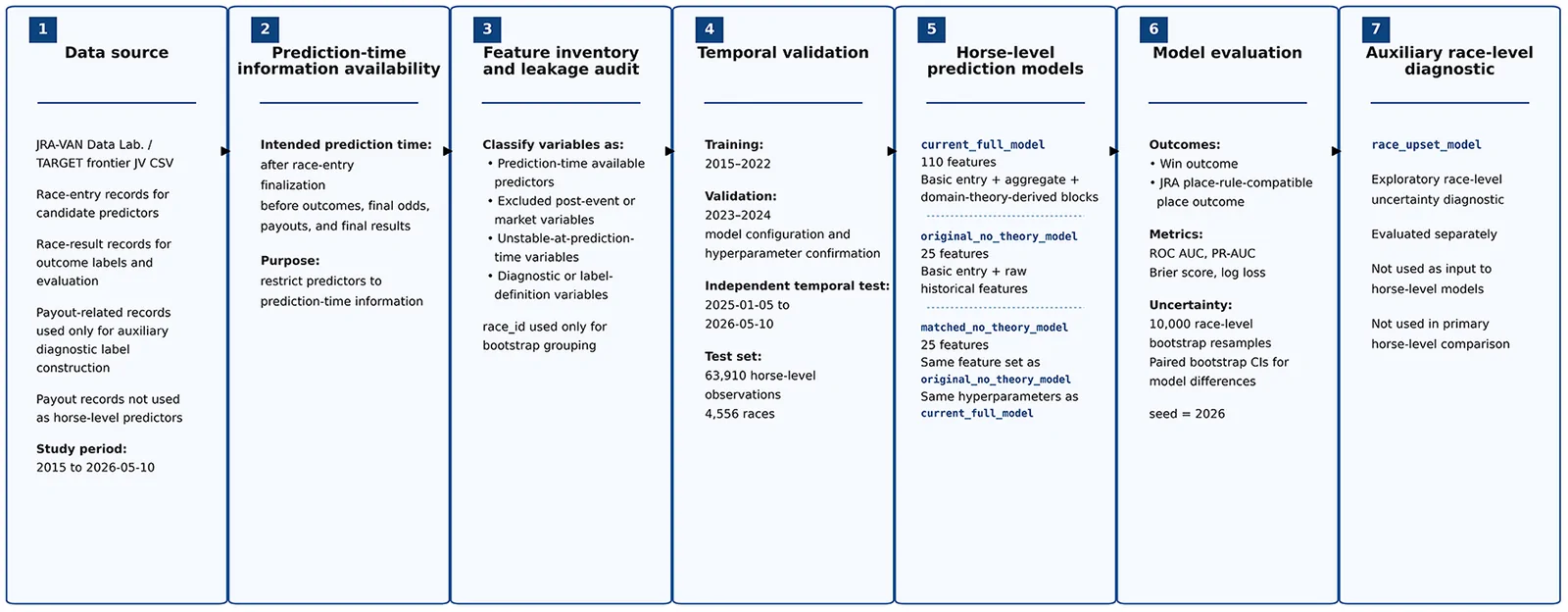
Leakage-aware evaluation framework defined by prediction-time information availability for prospective horse-race prediction under temporal validation. Candidate predictors were restricted to information available after race-entry finalization and before race outcomes, final odds, payouts, and final race results were known. Payout-related records were not used as horse-level predictive features. Variables were classified through a feature inventory and leakage audit into prediction-time available predictors, excluded post-event or market variables, variables with unstable availability at the intended prediction time, and diagnostic or outcome-label definition variables. Horse-level models were evaluated on an independent temporal test set using ROC AUC, PR-AUC, Brier score, and log loss, with race-level bootstrap confidence intervals and paired bootstrap confidence intervals for model differences. The race_upset_model was evaluated separately as an exploratory race-level uncertainty diagnostic and was not used as input to the horse-level prediction models or incorporated into the principal performance comparisons.

## 2 Materials and methods

### 2.1 Data source and temporal validation

Japanese Racing Association (JRA) flat-race data were obtained through JRA-VAN Data Lab (JRA-VAN, 2026) and exported in CSV format using TARGET frontier JV. The dataset included race-entry information used for candidate predictors, race-result records used for outcome-label construction and evaluation, and payout-related records used only for auxiliary diagnostic label construction. Payout records were not used as horse-level predictive features. Data from January 2015 through May 10, 2026 were used for analysis.

A temporal validation design was adopted. Data from 2015–2022 were used for model training, data from 2023–2024 were used for validation, and races conducted between January 5, 2025 and May 10, 2026 were reserved as an independent test set. The validation period was used for intermediate model assessment, model-configuration checks, and confirmation of hyperparameter settings. Final performance reporting and model comparison were based on the independent temporally separated test set. The test set consisted of 63,910 horse-level observations from 4,556 races. Because temporal dependence may lead to overly optimistic estimates when random train–test splitting is used, an independent future test set was employed to evaluate model generalization performance (Roberts et al., 2017).

### 2.2 Pre-event information constraints and leakage audit

The intended prediction time was after race-entry finalization and before race outcomes were known. Predictive features were restricted to information considered available at that point in the JRA-VAN/TARGET frontier JV data environment. Included variables comprised racecourse, course type, race distance, draw position, horse number, sex, age, assigned weight, jockey, trainer, pedigree information, and historical features derived exclusively from races preceding the target race.

Final odds, popularity rankings, finishing positions, payouts, race outcomes, final 3-furlong times, and information derived from outcomes within the same race were excluded because these variables are unavailable before outcomes are known or may reflect post-event information. Same-day body weight, going, and weather variables were also excluded because their availability or stability at the intended prediction time was considered insufficient in the target data environment.

A feature inventory and leakage audit were conducted to classify variables into prediction-time available variables, prediction-time unavailable variables, variables with unstable prediction-time availability, identifier variables, and variables used only for outcome-label definition. The variables race_id and horse_id were not used as predictive covariates. race_id was used only as the grouping variable for bootstrap resampling. The leakage audit was designed to verify that no information unavailable during deployment was incorporated into model development or evaluation procedures (Kaufman et al., 2012). The complete feature inventory and model-inclusion table are provided in Supplementary Table S1. Table 1 summarizes the feature and leakage inventory used in this study.

**Table 1:** Feature and leakage inventory.

Category	Example variables or features	Use in this study	Rationale
Basic entry information	Racecourse, surface type, distance, distance category, race number, horse number, gate number, sex, age, assigned weight, jockey, trainer, sire, damsire, field size	Included	Considered available after race-entry finalization
Historical features prior to the target race	starts_before, days_since_prev, win_rate_hist, place_rate_hist_jra, mean_finish_last3, hist_distance_change _from_prev, hist_surface_changed _from_prev	Used_in original_no_theory _model and matched_no_theory _model	Constructed exclusively from information available before the target race and therefore suitable for deployment-time prediction
Aggregate and domain-derived features	Jockey aggregate features, sire aggregate features, same-course domain-theory features, maiden-venue domain-theory features, rule-based flags	Used in current_full _model	Evaluated as aggregate feature blocks and domain-derived feature blocks
Post-event information	Finishing position, payouts, race results, final 3F time, outcome information from the same race	Excluded	Observed after the prediction time and may introduce leakage
Market and outcome-related information	Final odds, final popularity rank	Excluded	Treated as information finalized after the intended prediction time
Information with unstable availability	Same-day body weight, going, and weather	Excluded	Availability and stability may vary at the intended prediction time
Identifiers	race_id, horse_id	Excluded from predictive features	Not intended as predictive covariates; race_id was used only as the grouping variable for bootstrap resampling
Information used for place-label definition	Processed field_size	Used for defining y_place_jra; field-size information was also included as a race-entry-level predictor when available at prediction time	The place-label implementation used the processed field-size variable exported by TARGET frontier JV; this may reflect the final starter count and should not be interpreted as an exact reconstruction of every real-time betting condition

### 2.3 Feature sets and model development

Three horse-level prediction models were evaluated: current_full_model, an augmented feature model including basic entry features, aggregate feature blocks, and domain-theory-derived feature blocks; original_no_theory_model, the original comparison model using basic entry and raw historical features while excluding aggregate and domain-theory-derived feature blocks; and matched_no_theory_model, a sensitivity-analysis model using the same 25-feature set as original no theory model and the same HistGradientBoostingClassifier hyperparameters as current_full_model.

The current_full_model consisted of 110 features, including basic entry features, aggregate feature blocks, and domain-theory-derived feature blocks. These feature groups included jockey aggregate features, sire aggregate features, same-course domain-theory-derived features, maiden-venue domain-theory-derived features, and rule-based flags. These blocks were constructed from race-entry information and historical information available before the target race.

The original_no_theory_model consisted of 25 features based on basic entry features and raw historical features, excluding aggregate and domain-theory-derived feature blocks.

The matched_no_theory_model used the same 25-feature set as original_no_theory_model but applied the same HistGradientBoostingClassifier hyperparameters as current_full_model. This model was introduced as a hyperparameter-matched sensitivity-analysis model to help distinguish the influence of feature composition from differences in hyperparameter settings.

The primary comparison in this study was between current_full_model, which incorporated aggregate and domain-theory-derived feature blocks, and matched_no_theory_model, which excluded these feature groups while using identical hyperparameter settings.

Because feature design is known to substantially influence predictive performance (Guyon and Elisseeff, 2003), explicit comparisons were conducted between the augmented feature representation and the simpler no-theory historical feature representation.

All models were trained using HistGradientBoostingClassifier from scikit-learn (Pedregosa et al., 2011). Separate models were developed for the win binary outcome and the JRA place-rule-compatible binary place outcome. Their corresponding internal binary label names were y_win and y_place_jra, respectively. HistGradientBoostingClassifier hyperparameters are provided in Supplementary Table S2.

### 2.4 Outcomes

The primary horse-level binary outcome labels were the win outcome and the JRA place-rule-compatible place outcome. The corresponding internal binary label names were y_win and y_place_jra. The win outcome indicated whether a horse won the target race. The JRA place-rule-compatible place outcome indicated whether a horse finished within the payout-eligible place range under JRA place-betting rules (Japan Racing Association, 2026).

When used as a predictor, field_size was treated as a race-entry-level field-size variable available at the intended prediction time. For outcome-label construction, the processed field_size variable from the TARGET frontier JV race-result export was used to implement the JRA place-rule-compatible place outcome. Horses finishing within the top three positions were labeled positive when processed field size was eight or greater, whereas horses finishing within the top two positions were labeled positive when processed field_size ranged from five to seven. Races with four or fewer starters were excluded from place-outcome evaluation. Because the processed field_size variable may reflect the final starter count after scratches or entry changes, this outcome should be interpreted as a processed-data implementation of the JRA place rule rather than as a reconstruction of every real-time betting condition.

### 2.5 Software environment

The analysis pipeline was implemented in Python using pandas, NumPy, and scikit-learn. The retained model objects were loadable without version-mismatch warnings in the local revision environment using Python 3.13.7, scikit-learn 1.8.0, pandas 3.0.2, and NumPy 2.4.4. The exact Python version used when the original submitted model artifacts were fitted was not stored in the model metadata and therefore could not be retrospectively verified.

### 2.6 Performance evaluation

Model performance was assessed using the area under the receiver operating characteristic curve (ROC AUC), precision–recall area under the curve (PR-AUC), Brier score, and log loss.

Ninety-five percent confidence intervals were estimated using 10,000 race-level non-parametric bootstrap resamples based on race_id. The random seed was fixed at 2026. In addition to model-specific bootstrap confidence intervals, paired race-level bootstrap confidence intervals for model-performance differences were calculated on the independent test set. Validation-set performance is reported in Supplementary Table S4, whereas paired model-difference estimates are reported in Supplementary Table S3.

In addition to ROC AUC and PR-AUC, Brier score and log loss were evaluated to assess the quality of predicted probabilities. These metrics provide complementary information regarding probabilistic prediction performance (Gneiting and Raftery, 2007).

### 2.7 Auxiliary race-level diagnostic

In addition to horse-level prediction models, race_upset_model was evaluated as an exploratory and auxiliary race-level uncertainty diagnostic. The diagnostic target was constructed at the race level from historical post-event race outcomes, including payout-related and favorite-performance patterns. These post-event variables were used only to define the diagnostic race-level label and were not used as horse-level predictive features.

The predictor set for race_upset_model was restricted to pre-event or model-output summaries available before race outcomes were known. The model was trained and evaluated separately from the horse-level prediction models. Its outputs were not used for training, scoring, ranking, or evaluating the horse-level win and place prediction models. Accordingly, race_upset_model should be interpreted as an exploratory race-level uncertainty diagnostic rather than as part of the primary confirmatory horse-level prediction analysis.

## 3 Results

### 3.1 Test-set composition and leakage audit

The test set consisted of JRA flat races conducted between January 5, 2025 and May 10, 2026 and included 63,910 horse-level observations from 4,556 races.

For evaluation of the JRA place-rule-compatible place outcome, analyses were restricted to races with a processed field_size of at least five starters, resulting in 63,481 eligible horse-level observations. Among these, 13,567 observations were positive cases. A total of 116 observations differed between the JRA place-rule-compatible place outcome and a simple top-three-finishing definition.

The feature inventory and leakage audit confirmed that final odds, popularity rankings, finishing positions, payouts, race outcomes, final 3-furlong times, outcome information from the same race, same-day body weight, going, weather, race_id, and horse_id were not included as predictive features. All raw historical features were derived exclusively from information available prior to the target race.

### 3.2 Main horse-level model performance

Horse-level prediction performance is summarized in Table 2.

**Table 2:** Main horse-level model performance with 10,000 race_id-level bootstrap CIs.

Outcome	Model	ROC AUC	PR-AUC	Brier score	Log loss
Win outcome	current_full_model	0.7293 [0.7224, 0.7362]	0.1779 [0.1709, 0.1859]	0.0628 [0.0623, 0.0632]	0.2340 [0.2323, 0.2356]
Win outcome	original_no_theory_model	0.7507 [0.7440, 0.7573]	0.1939 [0.1863, 0.2024]	0.0621 [0.0616, 0.0625]	0.2298 [0.2281, 0.2314]
Win outcome	matched_no_theory_model	0.7543 [0.7475, 0.7609]	0.1974 [0.1891, 0.2060]	0.0619 [0.0615, 0.0624]	0.2287 [0.2269, 0.2306]
JRA place-rule-compatible place outcome	current_full_model	0.7164 [0.7118, 0.7212]	0.4159 [0.4087, 0.4235]	0.1501 [0.1492, 0.1511]	0.4668 [0.4642, 0.4693]
JRA place-rule-compatible place outcome	original_no_theory_model	0.7465 [0.7421, 0.7510]	0.4513 [0.4436, 0.4592]	0.1454 [0.1444, 0.1463]	0.4525 [0.4500, 0.4550]
JRA place-rule-compatible place outcome	matched_no_theory_model	0.7513 [0.7469, 0.7558]	0.4532 [0.4455, 0.4611]	0.1443 [0.1433, 0.1453]	0.4483 [0.4455, 0.4510]

For the win outcome, the ROC AUC was 0.7293 (95% CI: 0.7224–0.7362) for current_full_model, 0.7507 (95% CI: 0.7440–0.7573) for original_no_theory_model, and 0.7543 (95% CI: 0.7475–0.7609) for matched_no_theory_model.

For the JRA place-rule-compatible place outcome, the ROC AUC was 0.7164 (95% CI: 0.7118–0.7212) for current_full_model, 0.7465 (95% CI: 0.7421–0.7510) for original_no_theory_model, and 0.7513 (95% CI: 0.7469–0.7558) for matched_no_theory_model.

The matched_no_theory_model achieved the highest PR-AUC values for both binary labels. In addition, it achieved the lowest Brier scores and log-loss values among the evaluated horse-level models. Complete performance metrics are reported in Table 2.

### 3.3 Sensitivity analysis

Although current_full_model and original_no_theory_model were based on the same model family, their hyperparameter settings were not fully identical.

To assess whether the observed performance differences could be attributed solely to hyperparameter selection, the matched_no_theory_model was evaluated using the same model hyperparameters as the current_full_model.

Across both outcomes, the matched_no_theory_model demonstrated performance that was comparable to or better than that of the original_no_theory_model and consistently outperformed the current full model across all primary evaluation metrics.

Validation-set performance showed the same overall pattern and is reported in Supplementary Table S4. Paired race-level bootstrap analyses of test-set model differences are reported in Supplementary Table S3.

### 3.4 Auxiliary race-level uncertainty diagnostic

race_upset_model was evaluated as an exploratory and auxiliary race-level uncertainty diagnostic (Table 3).

**Table 3:** Auxiliary race_upset_model diagnostic performance with 10,000 race_id-level bootstrap CIs.

Model	ROC AUC	PR-AUC	Brier score	Log loss
race_upset_model	0.6464 [0.6304, 0.6624]	0.6224 [0.6000, 0.6449]	0.2327 [0.2294, 0.2361]	0.6571 [0.6499, 0.6642]

Across the 4,556 races in the test set, the model achieved a ROC AUC of 0.6464 (95% CI: 0.6304–0.6624), a PR-AUC of 0.6224 (95% CI: 0.6000–0.6449), a Brier score of 0.2327 (95% CI: 0.2294–0.2361), and a log loss of 0.6571 (95% CI: 0.6499–0.6642).

The outputs of race_upset_model were not incorporated into the horse-level prediction models.

### 3.5 Summary of findings

The primary findings were consistent when evaluated using the JRA place-rule-compatible place outcome and the corresponding historical feature (place_rate_hist_jra).

The no-theory feature set, consisting of basic entry features and raw historical features, achieved better predictive performance than the feature set incorporating aggregate feature blocks and domain-derived feature blocks used in current_full_model.

Under the temporally separated test set used in this study, the evaluated theory-score block did not provide additional improvements in predictive performance.

## 4 Discussion

The present study developed and evaluated a leakage-aware machine-learning evaluation framework for prospective horse-race prediction using only information considered available after race-entry finalization and before race outcomes were known. The methodological contribution is the integration of prediction-time information availability, feature inventory, leakage audit, temporal validation, feature-block comparison, matched sensitivity analysis, and race-level bootstrap uncertainty estimation into a single evaluation workflow. Two principal findings emerged. First, the no-theory feature representation used by original_no_theory_model and matched_no_theory_model outperformed the augmented feature representation used by current_full_model. Second, these findings remained consistent under temporally separated evaluation and race-level bootstrap uncertainty estimation, suggesting that the observed performance differences were not attributable to a particular train–test split.

One possible explanation for the inferior performance of the evaluated theory-score block is that additional feature engineering does not necessarily provide independent predictive information beyond that already contained in basic entry information and historical performance variables. Aggregate and domain-derived features may also introduce noise, redundancy, or reduced robustness when applied to future data. Because the present study was designed as an empirical evaluation rather than a causal analysis of feature utility, the specific mechanisms underlying this result cannot be determined from the current data alone. Nevertheless, the findings indicate that greater feature complexity did not translate into improved predictive performance under the deployment-oriented evaluation framework used in this study.

Importantly, these results should not be interpreted as evidence against the usefulness of domain knowledge in general. Domain expertise remains essential for identifying candidate predictors, defining outcomes, designing validation strategies, and interpreting model outputs. Rather, the present findings suggest that domain-derived feature blocks should be evaluated incrementally and empirically through leakage audits, temporal validation, feature-block ablation studies, and matched sensitivity analyses before deployment. The discrepancy between intuitive feature value and observed predictive utility highlights the importance of rigorous evaluation procedures in machine-learning applications.

The broader implication of this study extends beyond horse-racing prediction. Similar challenges arise in sports analytics, event forecasting, financial prediction, and other prospective prediction tasks where information available during model development differs from information available at prediction time. Similar challenges have also been recognized in other deployment-oriented machine-learning applications, where prediction-time information availability, external validation, and continual model monitoring are important for reliable real-world implementation (Yuan, 2024). In such settings, prediction-time information constraints, leakage prevention, and temporally separated validation may be as important as model architecture or feature complexity. The framework presented here provides a practical approach for evaluating machine-learning systems under deployment-oriented conditions and may contribute to improving the reliability of real-world predictive models.

Several limitations should be acknowledged. First, the study focused exclusively on Japanese flat racing using data obtained through JRA-VAN Data Lab. and exported through TARGET frontier JV; therefore, the findings may not generalize directly to other racing jurisdictions, data providers, or prediction domains. Second, only the currently implemented theory-score block was evaluated, and alternative domain-derived feature constructions may yield different results. Third, although temporal validation reduces the risk of overly optimistic estimates, future changes in racing environments, participant behavior, or data collection practices could affect model performance. Fourth, feature-importance analyses and calibration curves were not used as primary evidence in this manuscript and should be examined in future work alongside additional feature-block ablation analyses. Future studies should evaluate additional feature representations, alternative learning algorithms, and external datasets while further examining the generalizability of leakage-aware evaluation frameworks across prospective prediction tasks. Improving reproducibility through transparent reporting of predictor definitions, model configurations, and validation procedures remains an important challenge for prediction-model research (Belbasis and Panagiotou, 2022).

## 5 Conclusion

This study developed and evaluated a leakage-aware machine-learning evaluation framework for prospective horse-race prediction using only information available after race-entry finalization. The framework combined feature inventories, leakage audits, temporal validation, feature-block ablation, matched sensitivity analyses, and race-level bootstrap uncertainty estimation to assess predictive performance under deployment-oriented conditions.

Within this framework, a simpler feature set based on basic entry features and raw historical features outperformed the evaluated domain-derived theory-score block on a temporally separated test set. However, this finding should not be interpreted as evidence against the value of domain knowledge itself. Instead, the results demonstrate the importance of empirically validating domain-derived feature blocks under realistic prediction-time constraints and leakage-aware evaluation procedures.

Although demonstrated using horse-racing prediction, the proposed framework may be applicable to a broader range of prospective prediction problems in which information availability changes over time and data leakage poses a risk to model validity. Emphasizing prediction-time availability, temporal validation, and transparent feature evaluation may improve the reliability and practical utility of machine-learning systems deployed in real-world settings.

## Data Availability

The data analyzed in this study are subject to the following licenses/restrictions: the datasets were derived from horse-racing records obtained through JRA-VAN Data Lab. and exported using TARGET frontier JV. Access to these data is governed by licensing agreements with the data providers. Consequently, neither the raw data nor the processed datasets can be publicly released, redistributed, or included in the manuscript or Supplementary Material. Feature definitions, model specifications, evaluation procedures, and aggregate analysis outputs are provided in the manuscript and Supplementary Material to the extent possible without including licensed data. Requests to access these datasets should be directed to JRA-VAN Data Lab. (https://jra-van.jp) and TARGET frontier JV (https://jra-van.jp/target/).

## References

[B1] Belbasis L. Panagiotou O. A. (2022). Reproducibility of prediction models in health services research. BMC Res. Notes 15:204. doi: 10.1186/s13104-022-06082-4 35690767 PMC9188254

[B2] Bolton R. N. Chapman R. G. (1986). Searching for positive returns at the track: a multinomial logit model for handicapping horse races. Manage. Sci. 32, 1040–1060. doi: 10.1287/mnsc.32.8.1040

[B3] Bunker R. P. Thabtah F. (2019). A machine learning framework for sport result prediction. Appl. Comput. Inf. 15, 27–33. doi: 10.1016/j.aci.2017.09.005

[B4] Collins G. S. Moons K. G. M. Dhiman P. Riley R. D. Beam A. L. Van Calster B. . (2024). TRIPOD+AI statement: updated guidance for reporting clinical prediction models that use regression or machine learning methods. BMJ 385:e078378. doi: 10.1136/bmj-2023-078378 38626948 PMC11019967

[B5] Gneiting T. Raftery A. E. (2007). Strictly proper scoring rules, prediction, and estimation. J. Am. Stat. Assoc. 102, 359–378. doi: 10.1198/016214506000001437

[B6] Guyon I. Elisseeff A. (2003). An introduction to variable and feature selection. J. Mach. Learn. Res. 3, 1157–1182.

[B7] Horvat T. Job J. (2020). The use of machine learning in sport outcome prediction: a review. WIREs Data Mining Knowl. Disc. 10:e1380. doi: 10.1002/widm.1380

[B8] Japan Racing Association (2026). Place betting rule information. Available online at: https://www.jra.go.jp/kouza/yougo/w432.html (Accessed May 31, 2026).

[B9] JRA-VAN (2026). JRA-VAN Data Lab. Available online at: https://jra-van.jp/dlb/ (Accessed May 31, 2026).

[B10] Kaufman S. Rosset S. Perlich C. Stitelman O. (2012). Leakage in data mining: Formulation, detection, and avoidance. ACM Trans. Knowl. Discov. Data 6, 1–21. doi: 10.1145/2382577.2382579

[B11] Lessmann S. Sung M.-C. Johnson J. E. V. (2009). Identifying winners of competitive events: a SVM-based classification model for horserace prediction. Eur. J. Oper. Res. 196, 569–577. doi: 10.1016/j.ejor.2008.03.018

[B12] Pedregosa F. Varoquaux G. Gramfort A. Michel V. Thirion B. Grisel O. . (2011). Scikit-learn: Machine Learning in Python. J. Mach. Learn. Res. 12, 2825–2830. Available online at: http://jmlr.org/papers/v12/pedregosa11a.html

[B13] Roberts D. R. Bahn V. Ciuti S. Boyce M. S. Elith J. Guillera-Arroita G. . (2017). Cross-validation strategies for data with temporal, spatial, hierarchical, or phylogenetic structure. Ecography 40, 913–929. doi: 10.1111/ecog.02881

[B14] Yuan H. (2024). Toward real-world deployment of machine learning for health care: External validation, continual monitoring, and randomized clinical trials. Health Care Sci. 3, 360–364. doi: 10.1002/hcs2.114 39479276 PMC11520244

