# Supplementary material for: Leakage-aware pre-event machine learning evaluation for horse-race prediction under temporal validation
Source: Front Artif Intell. 2026 Sep 24;9:1896192. doi: 10.3389/frai.2026.1896192 (PMC13646064; doi:10.3389/frai.2026.1896192)
Supplement: Supplementary file 1 [file Data_Sheet_1.pdf]

Supplementary Material: Leakage-aware pre-event machine learning evaluation for horse-race prediction under temporal validation

Shuichi Sugiura

This document is the official Supplementary PDF for the accepted manuscript. It provides the complete feature inventory, HistGradientBoostingClassifier hyperparameters, paired race-level bootstrap model-difference estimates, and validation-set performance as appendix tables.

Throughout the supplementary tables, outcomes are displayed using descriptive labels. The descriptive label "Win outcome" corresponds to the internal horse-level binary label `y_win`, and "JRA place-rule-compatible place outcome" corresponds to the internal horse-level binary label `y_place_jra`, which was derived using the processed `field_size` variable. These labels correspond to the outcome definitions described in the Methods section of the main manuscript.

Supplementary Table S1. Complete feature inventory and model inclusion

The complete feature inventory is split into two landscape long tables to avoid text overlap while preserving all requested fields. Long snake-case feature names are allowed to wrap after underscores. Supplementary Table S1a reports feature names, descriptions, and feature blocks. Supplementary Table S1b reports model inclusion and prediction-time availability/leakage classification for the same features.

Supplementary Table S1a. Feature descriptions and feature blocks

Feature name	Feature description	Feature block
course	Racecourse identifier.	Basic entry feature
race_no	Race number within the race day.	Basic entry feature
horse_no	Horse number in the race-entry table.	Basic entry feature
frame_no	Gate/frame number in the race-entry table.	Basic entry feature
sex	Horse sex as recorded before the race.	Basic entry feature
age	Horse age at the target race.	Basic entry feature
jockey	Jockey name or identifier.	Basic entry feature
jockey_code	Jockey code.	Basic entry feature
trainer	Trainer name or identifier.	Basic entry feature
trainer_code	Trainer code.	Basic entry feature
surface	Race surface type.	Basic entry feature
distance	Race distance in meters.	Basic entry feature
class_simple	Simplified race class label.	Basic entry feature
field_size	Field-size variable used as a race-entry-level predictor when available at prediction time; processed field size from the race-result export was used separately for place-label construction.	Basic entry feature
carried_weight	Assigned carried weight.	Basic entry feature
sire	Sire identifier/name.	Basic entry feature
damsire	Damsire identifier/name.	Basic entry feature
theory_same_racecourse_experience	Domain-derived same-course or same-racecourse experience flag.	Same-course/domain-derived feature
theory_same_course_experience	Domain-derived same-course or same-racecourse experience flag.	Same-course/domain-derived feature
theory_same_course_good_result	Domain-derived same-course or same-racecourse experience flag.	Same-course/domain-derived feature
theory_same_course_win_experience	Domain-derived same-course or same-racecourse experience flag.	Same-course/domain-derived feature
theory_age2_maiden_same_course	Domain-derived rule flag based on historical or race-entry information.	Same-course/domain-derived feature
theory_age2_1win_same_course	Domain-derived rule flag based on historical or race-entry information.	Same-course/domain-derived feature
theory_age3_maiden_same_course	Domain-derived rule flag based on historical or race-entry information.	Same-course/domain-derived feature
theory_age3_1win_same_course	Domain-derived rule flag based on historical or race-entry information.	Same-course/domain-derived feature
theory_age2_maiden_same_course_good_result	Domain-derived rule flag based on historical or race-entry information.	Same-course/domain-derived feature
theory_age2_1win_same_course_good_result	Domain-derived rule flag based on historical or race-entry information.	Same-course/domain-derived feature
theory_age3_maiden_same_course_good_result	Domain-derived rule flag based on historical or race-entry information.	Same-course/domain-derived feature
theory_age3_1win_same_course_good_result	Domain-derived rule flag based on historical or race-entry information.	Same-course/domain-derived feature
maiden_same_venue_best_finish	Domain-derived maiden or venue-related historical feature.	Maiden-venue domain-derived feature
maiden_same_venue_best_finish_cat	Domain-derived maiden or venue-related historical feature.	Maiden-venue domain-derived feature
maiden_same_venue_best_distance	Domain-derived maiden or venue-related historical feature.	Maiden-venue domain-derived feature
maiden_same_venue_best_surface	Domain-derived maiden or venue-related historical feature.	Maiden-venue domain-derived feature
maiden_same_venue_best_jockey	Domain-derived maiden or venue-related historical feature.	Maiden-venue domain-derived feature
maiden_same_venue_distance_change_cat	Domain-derived maiden or venue-related historical feature.	Maiden-venue domain-derived feature
maiden_same_venue_surface_change_cat	Domain-derived maiden or venue-related historical feature.	Maiden-venue domain-derived feature
maiden_same_venue_jockey_prev_cat	Domain-derived maiden or venue-related historical feature.	Maiden-venue domain-derived feature
maiden_same_venue_same_jockey_as_best_run	Domain-derived maiden or venue-related historical feature.	Maiden-venue domain-derived feature
theory_maiden_same_venue_experience	Domain-derived rule flag based on historical or race-entry information.	Same-course/domain-derived feature
theory_maiden_same_venue_best_2nd	Domain-derived rule flag based on historical or race-entry information.	Same-course/domain-derived feature
theory_maiden_same_venue_best_3rd	Domain-derived rule flag based on historical or race-entry information.	Same-course/domain-derived feature
theory_maiden_same_venue_best_4th	Domain-derived rule flag based on historical or race-entry information.	Same-course/domain-derived feature
theory_maiden_same_venue_best_5th	Domain-derived rule flag based on historical or race-entry information.	Same-course/domain-derived feature
theory_maiden_same_venue_best_6th	Domain-derived rule flag based on historical or race-entry information.	Same-course/domain-derived feature
theory_maiden_same_venue_best_7th_or_worse	Domain-derived rule flag based on historical or race-entry information.	Same-course/domain-derived feature
theory_maiden_same_venue_best_1st_exception	Domain-derived rule flag based on historical or race-entry information.	Same-course/domain-derived feature
theory_maiden_same_venue_distance_shortening	Domain-derived rule flag based on historical or race-entry information.	Same-course/domain-derived feature
theory_maiden_same_venue_same_distance	Domain-derived rule flag based on historical or race-entry information.	Same-course/domain-derived feature
theory_maiden_same_venue_distance_extension	Domain-derived rule flag based on historical or race-entry information.	Same-course/domain-derived feature
theory_maiden_same_venue_turf_to_dirt	Domain-derived rule flag based on historical or race-entry information.	Same-course/domain-derived feature
theory_maiden_same_venue_dirt_to_turf	Domain-derived rule flag based on historical or race-entry information.	Same-course/domain-derived feature
theory_maiden_same_venue_same_surface	Domain-derived rule flag based on historical or race-entry information.	Same-course/domain-derived feature
theory_maiden_same_venue_jockey_continue	Domain-derived rule flag based on historical or race-entry information.	Same-course/domain-derived feature
theory_maiden_same_venue_jockey_change	Domain-derived rule flag based on historical or race-entry information.	Same-course/domain-derived feature

Feature name	Feature description	Feature block
theory__maiden__same__venue__same__jockey__as__best__run	Domain-derived rule flag based on historical or race-entry information.	Same-course/domain-derived feature
theory__maiden__same__venue__good__run__2nd__or__3rd	Domain-derived rule flag based on historical or race-entry information.	Same-course/domain-derived feature
distance__band	Categorical distance band.	Basic entry feature
efg__jockey__racecourse__hist__n	Historical jockey aggregate feature computed before the target race.	Jockey aggregate feature
efg__jockey__racecourse__hist__place__sum	Historical jockey aggregate feature computed before the target race.	Jockey aggregate feature
efg__jockey__racecourse__place__mean	Historical jockey aggregate feature computed before the target race.	Jockey aggregate feature
efg__jockey__racecourse__place__lcb95	Historical jockey aggregate feature computed before the target race.	Jockey aggregate feature
theory__jockey__racecourse__lcb__high	Rule flag derived from historical jockey aggregate information.	Jockey aggregate feature
efg__jockey__surface__hist__n	Historical jockey aggregate feature computed before the target race.	Jockey aggregate feature
efg__jockey__surface__hist__place__sum	Historical jockey aggregate feature computed before the target race.	Jockey aggregate feature
efg__jockey__surface__place__mean	Historical jockey aggregate feature computed before the target race.	Jockey aggregate feature
efg__jockey__surface__place__lcb95	Historical jockey aggregate feature computed before the target race.	Jockey aggregate feature
theory__jockey__surface__lcb__high	Rule flag derived from historical jockey aggregate information.	Jockey aggregate feature
efg__jockey__course__surface__hist__n	Historical jockey aggregate feature computed before the target race.	Jockey aggregate feature
efg__jockey__course__surface__hist__place__sum	Historical jockey aggregate feature computed before the target race.	Jockey aggregate feature
efg__jockey__course__surface__place__mean	Historical jockey aggregate feature computed before the target race.	Jockey aggregate feature
efg__jockey__course__surface__place__lcb95	Historical jockey aggregate feature computed before the target race.	Jockey aggregate feature
theory__jockey__course__surface__lcb__high	Rule flag derived from historical jockey aggregate information.	Jockey aggregate feature
efg__jockey__course__distance__band__hist__n	Historical jockey aggregate feature computed before the target race.	Jockey aggregate feature
efg__jockey__course__distance__band__hist__place__sum	Historical jockey aggregate feature computed before the target race.	Jockey aggregate feature
efg__jockey__course__distance__band__place__mean	Historical jockey aggregate feature computed before the target race.	Jockey aggregate feature
efg__jockey__course__distance__band__place__lcb95	Historical jockey aggregate feature computed before the target race.	Jockey aggregate feature
theory__jockey__course__distance__band__lcb__high	Rule flag derived from historical jockey aggregate information.	Jockey aggregate feature
efg__jockey__sire__hist__n	Historical jockey aggregate feature computed before the target race.	Jockey aggregate feature
efg__jockey__sire__hist__place__sum	Historical jockey aggregate feature computed before the target race.	Jockey aggregate feature
efg__jockey__sire__place__mean	Historical jockey aggregate feature computed before the target race.	Jockey aggregate feature
efg__jockey__sire__place__lcb95	Historical jockey aggregate feature computed before the target race.	Jockey aggregate feature
theory__jockey__sire__lcb__high	Rule flag derived from historical jockey aggregate information.	Jockey aggregate feature
efg__jockey__sire__surface__hist__n	Historical jockey aggregate feature computed before the target race.	Jockey aggregate feature
efg__jockey__sire__surface__hist__place__sum	Historical jockey aggregate feature computed before the target race.	Jockey aggregate feature
efg__jockey__sire__surface__place__mean	Historical jockey aggregate feature computed before the target race.	Jockey aggregate feature
efg__jockey__sire__surface__place__lcb95	Historical jockey aggregate feature computed before the target race.	Jockey aggregate feature
theory__jockey__sire__surface__lcb__high	Rule flag derived from historical jockey aggregate information.	Jockey aggregate feature
efg__sire__surface__hist__n	Historical sire aggregate feature computed before the target race.	Sire aggregate feature
efg__sire__surface__hist__place__sum	Historical sire aggregate feature computed before the target race.	Sire aggregate feature
efg__sire__surface__place__mean	Historical sire aggregate feature computed before the target race.	Sire aggregate feature
efg__sire__surface__place__lcb95	Historical sire aggregate feature computed before the target race.	Sire aggregate feature
theory__sire__surface__lcb__high	Rule flag derived from historical sire aggregate information.	Sire aggregate feature
efg__sire__distance__band__hist__n	Historical sire aggregate feature computed before the target race.	Sire aggregate feature
efg__sire__distance__band__hist__place__sum	Historical sire aggregate feature computed before the target race.	Sire aggregate feature
efg__sire__distance__band__place__mean	Historical sire aggregate feature computed before the target race.	Sire aggregate feature
efg__sire__distance__band__place__lcb95	Historical sire aggregate feature computed before the target race.	Sire aggregate feature
theory__sire__distance__band__lcb__high	Rule flag derived from historical sire aggregate information.	Sire aggregate feature
efg__sire__course__surface__hist__n	Historical sire aggregate feature computed before the target race.	Sire aggregate feature
efg__sire__course__surface__hist__place__sum	Historical sire aggregate feature computed before the target race.	Sire aggregate feature
efg__sire__course__surface__place__mean	Historical sire aggregate feature computed before the target race.	Sire aggregate feature
efg__sire__course__surface__place__lcb95	Historical sire aggregate feature computed before the target race.	Sire aggregate feature
theory__sire__course__surface__lcb__high	Rule flag derived from historical sire aggregate information.	Sire aggregate feature
efg__sire__course__distance__band__hist__n	Historical sire aggregate feature computed before the target race.	Sire aggregate feature
efg__sire__course__distance__band__hist__place__sum	Historical sire aggregate feature computed before the target race.	Sire aggregate feature
efg__sire__course__distance__band__place__mean	Historical sire aggregate feature computed before the target race.	Sire aggregate feature
efg__sire__course__distance__band__place__lcb95	Historical sire aggregate feature computed before the target race.	Sire aggregate feature
theory__sire__course__distance__band__lcb__high	Rule flag derived from historical sire aggregate information.	Sire aggregate feature
efg__fit__count	Historical aggregate feature computed before the target race.	Aggregate/domain-derived feature
theory__efg__any__lcb__high	Domain-derived rule flag based on historical or race-entry information.	Aggregate/domain-derived feature
theory__efg__multi__lcb__high	Domain-derived rule flag based on historical or race-entry information.	Aggregate/domain-derived feature
starts__before	Number of starts before the target race.	Raw historical feature
days__since__prev	Days since the previous start before the target race.	Raw historical feature
win__rate__hist	Historical win rate before the target race.	Raw historical feature
place__rate__hist__jra	Historical JRA place-rule-compatible place rate before the target race.	Raw historical feature

Feature name	Feature description	Feature block
mean_finish_last3	Mean finishing position over up to the last three prior starts.	Raw historical feature
hist_distance_change_from_prev	Distance change from the previous start.	Raw historical feature
hist_surface_changed_from_prev	Indicator of surface change from the previous start.	Raw historical feature

Supplementary Table S1b. Feature-set inclusion and leakage classification

Feature name	current	original	matched	Prediction-time availability/leakage classification
course	Yes	Yes	Yes	Prediction-time available feature.
race_no	Yes	Yes	Yes	Prediction-time available feature.
horse_no	Yes	Yes	Yes	Prediction-time available feature.
frame_no	Yes	Yes	Yes	Prediction-time available feature.
sex	Yes	Yes	Yes	Prediction-time available feature.
age	Yes	Yes	Yes	Prediction-time available feature.
jockey	Yes	Yes	Yes	Prediction-time available feature.
jockey_code	Yes	Yes	Yes	Prediction-time available feature.
trainer	Yes	Yes	Yes	Prediction-time available feature.
trainer_code	Yes	Yes	Yes	Prediction-time available feature.
surface	Yes	Yes	Yes	Prediction-time available feature.
distance	Yes	Yes	Yes	Prediction-time available feature.
class_simple	Yes	Yes	Yes	Prediction-time available feature.
field_size	Yes	Yes	Yes	Prediction-time available race-entry-level feature; processed field size from the TARGET frontier JV race-result export was used separately for JRA place-rule-compatible place-label construction and may reflect the final starter count. The place label should therefore be interpreted as a processed-data implementation of the JRA place rule rather than as an exact reconstruction of every real-time betting condition.
carried_weight	Yes	Yes	Yes	Prediction-time available feature.
sire	Yes	Yes	Yes	Prediction-time available feature.
damsire	Yes	Yes	Yes	Prediction-time available feature.
theory_same_racecourse_experience	Yes	No	No	Prediction-time available aggregate/domain-derived feature computed from pre-event or prior-race information.
theory_same_course_experience	Yes	No	No	Prediction-time available aggregate/domain-derived feature computed from pre-event or prior-race information.
theory_same_course_good_result	Yes	No	No	Prediction-time available aggregate/domain-derived feature computed from pre-event or prior-race information.
theory_same_course_win_experience	Yes	No	No	Prediction-time available aggregate/domain-derived feature computed from pre-event or prior-race information.
theory_age2_maiden_same_course	Yes	No	No	Prediction-time available aggregate/domain-derived feature computed from pre-event or prior-race information.
theory_age2_1win_same_course	Yes	No	No	Prediction-time available aggregate/domain-derived feature computed from pre-event or prior-race information.
theory_age3_maiden_same_course	Yes	No	No	Prediction-time available aggregate/domain-derived feature computed from pre-event or prior-race information.
theory_age3_1win_same_course	Yes	No	No	Prediction-time available aggregate/domain-derived feature computed from pre-event or prior-race information.
theory_age2_maiden_same_course_good_result	Yes	No	No	Prediction-time available aggregate/domain-derived feature computed from pre-event or prior-race information.
theory_age2_1win_same_course_good_result	Yes	No	No	Prediction-time available aggregate/domain-derived feature computed from pre-event or prior-race information.
theory_age3_maiden_same_course_good_result	Yes	No	No	Prediction-time available aggregate/domain-derived feature computed from pre-event or prior-race information.
theory_age3_1win_same_course_good_result	Yes	No	No	Prediction-time available aggregate/domain-derived feature computed from pre-event or prior-race information.
maiden_same_venue_best_finish	Yes	No	No	Prediction-time available aggregate/domain-derived feature computed from pre-event or prior-race information.
maiden_same_venue_best_finish_cat	Yes	No	No	Prediction-time available aggregate/domain-derived feature computed from pre-event or prior-race information.
maiden_same_venue_best_distance	Yes	No	No	Prediction-time available aggregate/domain-derived feature computed from pre-event or prior-race information.
maiden_same_venue_best_surface	Yes	No	No	Prediction-time available aggregate/domain-derived feature computed from pre-event or prior-race information.
maiden_same_venue_best_jockey	Yes	No	No	Prediction-time available aggregate/domain-derived feature computed from pre-event or prior-race information.
maiden_same_venue_distance_change_cat	Yes	No	No	Prediction-time available aggregate/domain-derived feature computed from pre-event or prior-race information.

Feature name	current	original	matched	Prediction-time availability/leakage classification
maiden__same__venue__surface__change__cat	Yes	No	No	Prediction-time available aggregate/domain-derived feature computed from pre-event or prior-race information.
maiden__same__venue__jockey__prev__cat	Yes	No	No	Prediction-time available aggregate/domain-derived feature computed from pre-event or prior-race information.
maiden__same__venue__same__jockey__as__best__run	Yes	No	No	Prediction-time available aggregate/domain-derived feature computed from pre-event or prior-race information.
theory__maiden__same__venue__experience	Yes	No	No	Prediction-time available aggregate/domain-derived feature computed from pre-event or prior-race information.
theory__maiden__same__venue__best__2nd	Yes	No	No	Prediction-time available aggregate/domain-derived feature computed from pre-event or prior-race information.
theory__maiden__same__venue__best__3rd	Yes	No	No	Prediction-time available aggregate/domain-derived feature computed from pre-event or prior-race information.
theory__maiden__same__venue__best__4th	Yes	No	No	Prediction-time available aggregate/domain-derived feature computed from pre-event or prior-race information.
theory__maiden__same__venue__best__5th	Yes	No	No	Prediction-time available aggregate/domain-derived feature computed from pre-event or prior-race information.
theory__maiden__same__venue__best__6th	Yes	No	No	Prediction-time available aggregate/domain-derived feature computed from pre-event or prior-race information.
theory__maiden__same__venue__best__7th__or__worse	Yes	No	No	Prediction-time available aggregate/domain-derived feature computed from pre-event or prior-race information.
theory__maiden__same__venue__best__1st__exception	Yes	No	No	Prediction-time available aggregate/domain-derived feature computed from pre-event or prior-race information.
theory__maiden__same__venue__distance__shortening	Yes	No	No	Prediction-time available aggregate/domain-derived feature computed from pre-event or prior-race information.
theory__maiden__same__venue__same__distance	Yes	No	No	Prediction-time available aggregate/domain-derived feature computed from pre-event or prior-race information.
theory__maiden__same__venue__distance__extension	Yes	No	No	Prediction-time available aggregate/domain-derived feature computed from pre-event or prior-race information.
theory__maiden__same__venue__turf__to__dirt	Yes	No	No	Prediction-time available aggregate/domain-derived feature computed from pre-event or prior-race information.
theory__maiden__same__venue__dirt__to__turf	Yes	No	No	Prediction-time available aggregate/domain-derived feature computed from pre-event or prior-race information.
theory__maiden__same__venue__same__surface	Yes	No	No	Prediction-time available aggregate/domain-derived feature computed from pre-event or prior-race information.
theory__maiden__same__venue__jockey__continue	Yes	No	No	Prediction-time available aggregate/domain-derived feature computed from pre-event or prior-race information.
theory__maiden__same__venue__jockey__change	Yes	No	No	Prediction-time available aggregate/domain-derived feature computed from pre-event or prior-race information.
theory__maiden__same__venue__same__jockey__as__best__run	Yes	No	No	Prediction-time available aggregate/domain-derived feature computed from pre-event or prior-race information.
theory__maiden__same__venue__good__run__2nd__or__3rd	Yes	No	No	Prediction-time available aggregate/domain-derived feature computed from pre-event or prior-race information.
distance__band	Yes	Yes	Yes	Prediction-time available feature.
efg__jockey__racecourse__hist__n	Yes	No	No	Prediction-time available aggregate/domain-derived feature computed from pre-event or prior-race information.
efg__jockey__racecourse__hist__place__sum	Yes	No	No	Prediction-time available aggregate/domain-derived feature computed from pre-event or prior-race information.
efg__jockey__racecourse__place__mean	Yes	No	No	Prediction-time available aggregate/domain-derived feature computed from pre-event or prior-race information.
efg__jockey__racecourse__place__lcb95	Yes	No	No	Prediction-time available aggregate/domain-derived feature computed from pre-event or prior-race information.
theory__jockey__racecourse__lcb__high	Yes	No	No	Prediction-time available aggregate/domain-derived feature computed from pre-event or prior-race information.
efg__jockey__surface__hist__n	Yes	No	No	Prediction-time available aggregate/domain-derived feature computed from pre-event or prior-race information.
efg__jockey__surface__hist__place__sum	Yes	No	No	Prediction-time available aggregate/domain-derived feature computed from pre-event or prior-race information.
efg__jockey__surface__place__mean	Yes	No	No	Prediction-time available aggregate/domain-derived feature computed from pre-event or prior-race information.
efg__jockey__surface__place__lcb95	Yes	No	No	Prediction-time available aggregate/domain-derived feature computed from pre-event or prior-race information.

Feature name	current	original	matched	Prediction-time availability/leakage classification
theory_jockey_surface_lcb_high	Yes	No	No	Prediction-time available aggregate/domain-derived feature computed from pre-event or prior-race information.
efg_jockey_course_surface_hist_n	Yes	No	No	Prediction-time available aggregate/domain-derived feature computed from pre-event or prior-race information.
efg_jockey_course_surface_hist_place_sum	Yes	No	No	Prediction-time available aggregate/domain-derived feature computed from pre-event or prior-race information.
efg_jockey_course_surface_place_mean	Yes	No	No	Prediction-time available aggregate/domain-derived feature computed from pre-event or prior-race information.
efg_jockey_course_surface_place_lcb95	Yes	No	No	Prediction-time available aggregate/domain-derived feature computed from pre-event or prior-race information.
theory_jockey_course_surface_lcb_high	Yes	No	No	Prediction-time available aggregate/domain-derived feature computed from pre-event or prior-race information.
efg_jockey_course_distance_band_hist_n	Yes	No	No	Prediction-time available aggregate/domain-derived feature computed from pre-event or prior-race information.
efg_jockey_course_distance_band_hist_place_sum	Yes	No	No	Prediction-time available aggregate/domain-derived feature computed from pre-event or prior-race information.
efg_jockey_course_distance_band_place_mean	Yes	No	No	Prediction-time available aggregate/domain-derived feature computed from pre-event or prior-race information.
efg_jockey_course_distance_band_place_lcb95	Yes	No	No	Prediction-time available aggregate/domain-derived feature computed from pre-event or prior-race information.
theory_jockey_course_distance_band_lcb_high	Yes	No	No	Prediction-time available aggregate/domain-derived feature computed from pre-event or prior-race information.
efg_jockey_sire_hist_n	Yes	No	No	Prediction-time available aggregate/domain-derived feature computed from pre-event or prior-race information.
efg_jockey_sire_hist_place_sum	Yes	No	No	Prediction-time available aggregate/domain-derived feature computed from pre-event or prior-race information.
efg_jockey_sire_place_mean	Yes	No	No	Prediction-time available aggregate/domain-derived feature computed from pre-event or prior-race information.
efg_jockey_sire_place_lcb95	Yes	No	No	Prediction-time available aggregate/domain-derived feature computed from pre-event or prior-race information.
theory_jockey_sire_lcb_high	Yes	No	No	Prediction-time available aggregate/domain-derived feature computed from pre-event or prior-race information.
efg_jockey_sire_surface_hist_n	Yes	No	No	Prediction-time available aggregate/domain-derived feature computed from pre-event or prior-race information.
efg_jockey_sire_surface_hist_place_sum	Yes	No	No	Prediction-time available aggregate/domain-derived feature computed from pre-event or prior-race information.
efg_jockey_sire_surface_place_mean	Yes	No	No	Prediction-time available aggregate/domain-derived feature computed from pre-event or prior-race information.
efg_jockey_sire_surface_place_lcb95	Yes	No	No	Prediction-time available aggregate/domain-derived feature computed from pre-event or prior-race information.
theory_jockey_sire_surface_lcb_high	Yes	No	No	Prediction-time available aggregate/domain-derived feature computed from pre-event or prior-race information.
efg_sire_surface_hist_n	Yes	No	No	Prediction-time available aggregate/domain-derived feature computed from pre-event or prior-race information.
efg_sire_surface_hist_place_sum	Yes	No	No	Prediction-time available aggregate/domain-derived feature computed from pre-event or prior-race information.
efg_sire_surface_place_mean	Yes	No	No	Prediction-time available aggregate/domain-derived feature computed from pre-event or prior-race information.
efg_sire_surface_place_lcb95	Yes	No	No	Prediction-time available aggregate/domain-derived feature computed from pre-event or prior-race information.
theory_sire_surface_lcb_high	Yes	No	No	Prediction-time available aggregate/domain-derived feature computed from pre-event or prior-race information.
efg_sire_distance_band_hist_n	Yes	No	No	Prediction-time available aggregate/domain-derived feature computed from pre-event or prior-race information.
efg_sire_distance_band_hist_place_sum	Yes	No	No	Prediction-time available aggregate/domain-derived feature computed from pre-event or prior-race information.
efg_sire_distance_band_place_mean	Yes	No	No	Prediction-time available aggregate/domain-derived feature computed from pre-event or prior-race information.
efg_sire_distance_band_place_lcb95	Yes	No	No	Prediction-time available aggregate/domain-derived feature computed from pre-event or prior-race information.
theory_sire_distance_band_lcb_high	Yes	No	No	Prediction-time available aggregate/domain-derived feature computed from pre-event or prior-race information.

Feature name	current	original	matched	Prediction-time availability/leakage classification
efg_sire_course_surface_hist_n	Yes	No	No	Prediction-time available aggregate/domain-derived feature computed from pre-event or prior-race information.
efg_sire_course_surface_hist_place_sum	Yes	No	No	Prediction-time available aggregate/domain-derived feature computed from pre-event or prior-race information.
efg_sire_course_surface_place_mean	Yes	No	No	Prediction-time available aggregate/domain-derived feature computed from pre-event or prior-race information.
efg_sire_course_surface_place_lcb95	Yes	No	No	Prediction-time available aggregate/domain-derived feature computed from pre-event or prior-race information.
theory_sire_course_surface_lcb_high	Yes	No	No	Prediction-time available aggregate/domain-derived feature computed from pre-event or prior-race information.
efg_sire_course_distance_band_hist_n	Yes	No	No	Prediction-time available aggregate/domain-derived feature computed from pre-event or prior-race information.
efg_sire_course_distance_band_hist_place_sum	Yes	No	No	Prediction-time available aggregate/domain-derived feature computed from pre-event or prior-race information.
efg_sire_course_distance_band_place_mean	Yes	No	No	Prediction-time available aggregate/domain-derived feature computed from pre-event or prior-race information.
efg_sire_course_distance_band_place_lcb95	Yes	No	No	Prediction-time available aggregate/domain-derived feature computed from pre-event or prior-race information.
theory_sire_course_distance_band_lcb_high	Yes	No	No	Prediction-time available aggregate/domain-derived feature computed from pre-event or prior-race information.
efg_fit_count	Yes	No	No	Prediction-time available aggregate/domain-derived feature computed from pre-event or prior-race information.
theory_efg_any_lcb_high	Yes	No	No	Prediction-time available aggregate/domain-derived feature computed from pre-event or prior-race information.
theory_efg_multi_lcb_high	Yes	No	No	Prediction-time available aggregate/domain-derived feature computed from pre-event or prior-race information.
starts_before	No	Yes	Yes	Prediction-time available historical feature derived only from races before the target race.
days_since_prev	No	Yes	Yes	Prediction-time available historical feature derived only from races before the target race.
win_rate_hist	No	Yes	Yes	Prediction-time available historical feature derived only from races before the target race.
place_rate_hist_jra	No	Yes	Yes	Prediction-time available historical feature derived only from races before the target race.
mean_finish_last3	No	Yes	Yes	Prediction-time available historical feature derived only from races before the target race.
hist_distance_change_from_prev	No	Yes	Yes	Prediction-time available historical feature derived only from races before the target race.
hist_surface_changed_from_prev	No	Yes	Yes	Prediction-time available historical feature derived only from races before the target race.

Supplementary Table S2. HistGradientBoostingClassifier hyperparameters

Hyperparameter	`current_full_model`	`original_no_theory_model`	`matched_no_theory_model`
categorical_features	from_dtype	from_dtype	from_dtype
class_weight	None	None	None
early_stopping	True	True	True
interaction_cst	None	None	None
l2_regularization	0.0	0.0	0.0
learning_rate	0.025	0.05	0.025
loss	log_loss	log_loss	log_loss
max_bins	255	255	255
max_depth	None	None	None
max_features	1.0	1.0	1.0
max_iter	1200	80	1200
max_leaf_nodes	63	31	63
min_samples_leaf	100	80	100
monotonic_cst	None	None	None
n_iter_no_change	50	10	50
random_state	42	42	42
scoring	loss	loss	loss
tol	1e-07	1e-07	1e-07
validation_fraction	0.1	0.1	0.1
verbose	0	0	0
warm_start	False	False	False

Supplementary Table S2 was regenerated from the retained model objects using the full HistGradientBoostingClassifier `get_params()` output for the estimator step. The retained `y_win` and `y_place_jra` model objects had identical HistGradientBoostingClassifier hyperparameter profiles within each model. The `matched_no_theory_model` used the same hyperparameter profile as `current_full_model`. The `original_no_theory_model` used the earlier no-theory baseline profile.

Supplementary Table S3. Paired bootstrap model differences

Race-level paired bootstrap model-difference estimates on the independent temporal test set are reported below.

Positive differences favor the first model for ROC AUC and PR-AUC. Negative differences favor the first model for Brier score and log loss. Outcome labels are displayed using descriptive names. The corresponding internal binary label names are `y_win` for the win outcome and `y_place_jra` for the JRA place-rule-compatible place outcome.

Comparison	Outcome	Metric	Difference	95% CI	Resamples
matched_no_theory_model - current_full_model	Win outcome	ROC AUC	0.0250	[0.0190, 0.0309]	10000
matched_no_theory_model - current_full_model	Win outcome	PR-AUC	0.0195	[0.0127, 0.0262]	10000
matched_no_theory_model - current_full_model	Win outcome	Brier score	-0.0008	[-0.0011, -0.0006]	10000
matched_no_theory_model - current_full_model	Win outcome	log loss	-0.0052	[-0.0065, -0.0040]	10000
original_no_theory_model - current_full_model	Win outcome	ROC AUC	0.0214	[0.0152, 0.0275]	10000
original_no_theory_model - current_full_model	Win outcome	PR-AUC	0.0161	[0.0094, 0.0226]	10000
original_no_theory_model - current_full_model	Win outcome	Brier score	-0.0007	[-0.0009, -0.0005]	10000
original_no_theory_model - current_full_model	Win outcome	log loss	-0.0042	[-0.0054, -0.0030]	10000
matched_no_theory_model - original_no_theory_model	Win outcome	ROC AUC	0.0036	[0.0016, 0.0057]	10000
matched_no_theory_model - original_no_theory_model	Win outcome	PR-AUC	0.0034	[0.0000, 0.0069]	10000
matched_no_theory_model - original_no_theory_model	Win outcome	Brier score	-0.0001	[-0.0002, 0.0000]	10000
matched_no_theory_model - original_no_theory_model	Win outcome	log loss	-0.0010	[-0.0015, -0.0005]	10000
matched_no_theory_model - current_full_model	JRA place-rule-compatible place outcome	ROC AUC	0.0350	[0.0310, 0.0389]	10000
matched_no_theory_model - current_full_model	JRA place-rule-compatible place outcome	PR-AUC	0.0373	[0.0310, 0.0435]	10000
matched_no_theory_model - current_full_model	JRA place-rule-compatible place outcome	Brier score	-0.0059	[-0.0066, -0.0051]	10000
matched_no_theory_model - current_full_model	JRA place-rule-compatible place outcome	log loss	-0.0185	[-0.0205, -0.0165]	10000
original_no_theory_model - current_full_model	JRA place-rule-compatible place outcome	ROC AUC	0.0302	[0.0259, 0.0343]	10000
original_no_theory_model - current_full_model	JRA place-rule-compatible place outcome	PR-AUC	0.0355	[0.0289, 0.0420]	10000
original_no_theory_model - current_full_model	JRA place-rule-compatible place outcome	Brier score	-0.0048	[-0.0055, -0.0041]	10000
original_no_theory_model - current_full_model	JRA place-rule-compatible place outcome	log loss	-0.0143	[-0.0162, -0.0123]	10000
matched_no_theory_model - original_no_theory_model	JRA place-rule-compatible place outcome	ROC AUC	0.0048	[0.0029, 0.0067]	10000
matched_no_theory_model - original_no_theory_model	JRA place-rule-compatible place outcome	PR-AUC	0.0018	[-0.0019, 0.0055]	10000
matched_no_theory_model - original_no_theory_model	JRA place-rule-compatible place outcome	Brier score	-0.0011	[-0.0015, -0.0007]	10000
matched_no_theory_model - original_no_theory_model	JRA place-rule-compatible place outcome	log loss	-0.0042	[-0.0052, -0.0032]	10000

Supplementary Table S4. Validation-set performance

Outcome	Model	Rows	Races	Features	ROC AUC	PR-AUC	Brier score	Log loss
Win outcome	current_full_model	92008	6656	110	0.7277	0.1801	0.0636	0.2366
JRA place-rule-compatible place outcome	current_full_model	91340	6656	110	0.7158	0.4129	0.1521	0.4711
Win outcome	original_no_theory_model	92008	6656	25	0.7557	0.2014	0.0627	0.2310
JRA place-rule-compatible place outcome	original_no_theory_model	91340	6656	25	0.7431	0.4465	0.1477	0.4585
Win outcome	matched_no_theory_model	92008	6656	25	0.7625	0.2053	0.0624	0.2290
JRA place-rule-compatible place outcome	matched_no_theory_model	91340	6656	25	0.7520	0.4538	0.1459	0.4522

The validation period covered 2023–2024 and was used for intermediate model assessment and model-configuration checks. Final conclusions were based on the independent temporally separated test set. The Outcome column uses descriptive labels; the corresponding internal binary label names are `y_win` for the win outcome and `y_place_jra` for the JRA place-rule-compatible place outcome.
